# The Doctor as Parent, Partner, Provider… or Comrade? Distribution of Power in Past and Present Models of the Doctor–Patient Relationship

**DOI:** 10.1007/s10728-021-00432-2

**Published:** 2021-04-27

**Authors:** Mani Shutzberg

**Affiliations:** Centre for Studies in Practical Knowledge, Södertörn University, Stockholm, Sweden

**Keywords:** Solidarity, Power, Doctor–patient relationship, Healthcare, Bureaucracy

## Abstract

The commonly occurring metaphors and models of the doctor–patient relationship can be divided into three clusters, depending on what distribution of power they represent: in the paternalist cluster, power resides with the physician; in the consumer model, power resides with the patient; in the partnership model, power is distributed equally between doctor and patient. Often, this tripartite division is accepted as an exhaustive typology of doctor–patient relationships. The main objective of this paper is to challenge this idea by introducing a fourth possibility and distribution of power, namely, the distribution in which power resides with neither doctor nor patient. This equality in powerlessness—the hallmark of “the age of bureaucratic parsimony”—is the point of departure for a qualitatively new doctor–patient relationship, which is best described in terms of *solidarity* between *comrades*. This paper specifies the characteristics of this specific type of solidarity and illustrates it with a case study of how Swedish doctors and patients interrelate in the sickness certification practice.

## Introduction: Metaphors and Models of the Doctor–Patient Relationship

This paper revisits the three main archetypes of the doctor–patient relationship. In them the doctor figures as *parent*, as *partner* and as (service) *provider*, respectively. The three clusters represent distinct distributions of power among the involved parties: in the first case power resides wholly in the role of the physician, in the second it is roughly equally divided between doctor and patient, and in the third it mainly rests in the hands of the patient-consumer. As we enter what the medical ethicist Mark Siegler calls the “age of bureaucratic parsimony” [54], a new type of doctor–patient relationship has become paradigmatic. This one is similar to the partnership model, insofar as power is distributed equally between doctor and patient. However, there is one perverted twist and decisive difference: In the age of bureaucratic parsimony, both doctor and patient are equally robbed of power and influence by instances outside of the immediate medical encounter. This new situation enables doctor and patient to interrelate as two similarly dominated subjects—as *comrades*. I suggest that an appropriate word for this new bond is *solidarity*.

This new medical bond is explored theoretically and with an empirical case, namely, the interaction and comportment of Swedish doctors and patients in the sickness certification process, which have changed drastically during the last years. The way doctors and patients act towards each other, and the way doctors resist the insurance agency, embodies this new kind of solidarity at the heart of the new doctor–patient relationship.

### The Physician as Parent

The grand narratives of the evolution of the doctor–patient relationship almost always start with what philosophers have referred to as “paternalism” since the 1960s and 1970s [6, 18]. The age of paternalism has been claimed to span between “many decades” [14] to “thousands of years” [54]. Etymologically, paternalism derives from the Latin word for father. This metaphor, then, aligns the relationship between doctor and patient with the relationship between benevolent father and ignorant child. The patient-child is regarded as a dependent of the doctor-father; just like the child of the father, the patient-child is not really actively involved in deliberations about his or her medical encounter. Belonging to the same cluster of metaphors is the so-called “priestly model” of doctor–patient interaction, which has designated more or less the same thing [63]. In terms of ethical principles, a paternalistic stance implies that the principle of beneficence often, or always, trumps the principle of patient autonomy [8, 62]. Thomas Szasz and Marc Hollender’s famous text about the doctor–patient relationship puts forth two distinct paternalist metaphors which correspond to two different types of doctor–patient interaction: the *parent-infant* and the *parent-adolescent* relationship. In the first, the doctor-parent is active, and the patient-infant is a completely passive recipient of the doctor’s actions. It is even questionable if it can be called “interaction” at all, as the action goes only one-way. It is a completely appropriate orientation in the treatment of emergencies, but not much else. The second parental metaphor, between *adult and adolescent* roughly designates a form of paternalism that partially recognizes patient subjectivity. In contrast to the infant, the adolescent is conscious. The adolescent can cooperate, or at least obey. Although critical of this model, Szasz and Hollender admit that there are scenarios when also this mode of interaction might be warranted, or at least commonly occurring: “it is employed in situations which are less desperate than those previously mentioned […]. Although the patient is ill, he is conscious and has feelings and aspirations of his own.” Their own suggested example of this is “acute infections” [57].

Despite the heterogeneity of the attitudes that huddle under the umbrella of paternalism and familial metaphors, there is one feature that unites them, and which is important for the progression of the main argument in this article. The doctor is the one in charge, with power to influence the decisions. The main characteristics “pertains to power, and to its actual or potential use. The more powerful of the two (parent, physician, employer, etc.) will expect […] cooperation of the other member of the pair (child, patient, employee, etc.). The patient is expected to ‘look up’ and to ‘obey’ his doctor” [57].

### The End of Paternalism, and the Start of Something New

The standard account of the doctor–patient relationship during the last century is a story of the abandonment of paternalism for something else [54, 57, 62]. The beginning of the end of the era of medical paternalism is often set around the end of the Second World War. Although the effective causes for the death of the hegemony of paternalism are notoriously difficult to pin-point, and separation between causes and effects are tricky, the changes in general attitudes are somewhat easier to follow. For example, an American survey in 1961 showed that 90% of the responding doctors preferred not to fully disclose a cancer diagnosis to their patients. About 20 years later, the response was completely reversed: 97% indicated a preference to tell their patients they have cancer [45]. For the medical ethicist Howard Brody, among others, this rapid change in information disclosure possibly represented a paradigmatic shift, leaving behind the “history of routine deception and withholding of information for fear that frank truth would frighten or harm the patient” [8].

### The Physician as Partner

There are several suggestions as to what superseded medical paternalism. One category and cluster of metaphors used to designate the new relationship emphasizes its equal and cooperative character [2, 15, 19, 63]. Doctor and patient are more like parties of a contract of sorts, both in the form of a business contract but also as a holy covenant, a marriage [63]; Others have suggested that the bond is analogous to friendship [19]. The common denominator of these models and metaphors can be summarized in a few points: 1. The flow of information between doctor and patient is more symmetric; less an interrogation and more a conversation; 2. An open deliberative approach to whatever means and ends are appropriate for the treatment of the patient; the ends of medicine are not presupposed, as in the paternalist case. In terms of power, they are all identical, as Szasz and Hollender put it: “It is crucial to this type of interaction that the participants […] have approximately equal power” [57]. A crucial common denominator for many who employ the metaphor (some cited above) is the relative indifference to power structures outside of the doctor–patient dyad. The power vested in doctors and patients is assumed to entail a high degree of actual influence over the ends of the medical with negligible apparent involvement by external forces. A corollary of this assumption is that the doctor and patient are the main (or sole) entities involved in deliberating and making decisions about the goals set in the confines of the doctor’s office. The negotiation is, hence, directed inwards [2, 8, 14, 15, 19, 48, 57, 63].

### The Physician as (Service) Provider

There is a third mode of doctor–patient relationship that has gained traction later than the partnership model: *the consumer model*.^1^ In the United States it can be traced back to the 1960s [7]. In a European welfare state context, the transition might have occurred later, during the (re-)commodification, privatization and marketization of healthcare around the 1980s (with considerable individual differences between countries, of course) [27].

Even though the model can be categorized as a realist offshoot of the partnership model, they differ in terms of power. The autonomous and sovereign patient-consumer constitutes the essential core of the metaphor. The professional role correlating to the patient-consumer is more passive: a provider or “engineer” [63], or a “seller” [7] of a service. At its most reified extreme, physicians *are* the services or objects they provide: mere products. Hence, the relationship is more asymmetrical than the partnership model, and can in some respects be conceived as the “polar opposite” of the paternalist model [7]. Analee and Thomas Beisecker have elegantly summarized the consumer metaphor in the doctor–patient relationship in the following bullet points: "Consumerism focuses on rights[;] consumerism assumes the doctor is self-centered[;] consumerism replaces trust with accountability[;] consumerism presumes that the patient's health care values dominate[;] consumerism may require third-party supervision" [7]. The fundamental assumption that forms the base of the consumer model is that the interests of the physician and the patient diverges, and the only person genuinely interested in the health of the patient is the patient. Most importantly, for the aims of this article, the specific distribution of power rests in the hands of the patient: “In a consumerist relationship, the seller has no particular authority; power rests in the buyer who can make the decisions to buy or not to buy as he or she sees fit “ [7].^2^

### Ordering the Metaphors

In working through the different metaphors of the doctor–patient relationship, I have implicitly ordered them in two ways. Firstly, there is the question of historical order in which the metaphors of the doctor–patient relationship appeared. Although there is good reason to be critical of grossly oversimplifying statements, I believe there is some truth to the specific order in which the paradigms are assumed to have appeared: that is, during the twentieth century in the West, paternalism was superseded by other models for the doctor–patient relationship, namely the partnership model and the consumer model.

Then there is the question of ordering the models and metaphors in terms of the specific power distribution they each represent. This categorization of models and metaphors is by no means an unprecedented endeavor. For example, Felicity Goodyear-Smith and Stephen Buetow have suggested something similar. In their meta-model, there are mainly three unique distributions of power: In paternalism power resides with the doctor, in consumerism with the patient, and in contemporary society both doctors and patients are empowered, but with “each exercising different but equally important sources of power”. They then proceed to problematize this last position of “equality” or “mutuality” of power. Both doctors and patients can misuse their powers; doctors have multiple allegiances to other stakeholders outside of the doctor–patient relationship which adds a good deal of complexity to the partnership model [24]. However, there is one possible configuration of power they do not explore, namely, the *disempowerment of both physician and patient* and the peculiar interaction it engenders.^3^

### The Equal Disempowerment of Physicians and Patients: Comradeship

The same historical and ideological conditions that brought forth the consumer model are also the ground for this fourth model: marketization, public sector cutbacks and a businesslike management of healthcare that remained public, all driven by a neoliberal frame of reference [38, 39]. The partnership model and the consumer model both presuppose that the unit of doctor and patient (regardless of the internal distribution of power) contains a high degree of control and power over the course of the medical encounter. Current conditions of power have in reality empowered neither doctor nor patient. Not only is the equal disempowerment of doctor and patient conceivable; in contemporary healthcare, I argue, the equal disempowerment of doctor and patient is becoming paradigmatic. As far back as 1985, and possibly a bit untimely, Mark Siegler declared the arrival of what he called the “age of bureaucratic parsimony” in healthcare. For Siegler, the distinctive feature of this age was that the relationship between doctor and patient had ceased to be the impenetrable dyadic bond it once was: “The physician will be accountable to multiple interests including employers (health maintenance organizations [HMOs], hospital corporations), hospitals, insurance companies, and government. The physician in the age of bureaucracy will have divided allegiances” [54]. The condition of possibility of the equal disempowerment of doctor and patient is this multiplication of stakeholders in healthcare, and that *power resides elsewhere*.

A warranted objection is that the doctor–patient relationship never existed in isolation, that external stakeholders and actors (such as family members or social insurance agencies) and more impersonal, abstract, power structures (such as class, culture, market, gender, etc.) always have conditioned and influenced the patient-doctor interaction [1, 35, 54, 58, 64]. These constitute a dimension of external force behind all the enumerated models, that can be large or small, depending on the circumstances. For example, does the partnership model not already contain the possibility of an even distribution of whatever power there is (be it a lot or very little), reducing a distinction between an even distribution of power and an even distribution of powerlessness to a category mistake? As mentioned above, characterizations of the partnership model usually exclude the possibility that powers of doctors and patients might be severely constrained by external forces. Secondly, the partnership model deserves a distinct category as its normative claims are laudable, despite its limited descriptive value in this particular conjuncture. Thirdly, although the partnership model and the model I propose both formally share an even distribution of power, the experiences they produce are qualitatively different. Whereas the partnership model mainly brings to the fore inward negotiation, my suggested equal disempowerment model is dominated by negotiation strategies directed towards the structures in which the doctor–patient interaction is embedded. Hence, another difference is that schematic partnership models allow a bracketing of outside forces without losing grasp of the interaction between doctor and patient as partners (be it for ideological or pedagogical reasons). The main characteristics of how disempowered doctors and patients act and interact, on the other hand, cannot even be comprehended without reference to its outside.

The novelty of my proposition, I believe, is that this relatively new condition of healthcare creates not only problems, but also possibilities. It is not *despite* the characteristics of this age, but *because of them*, that a new kind of bond can be established between doctors and patients. Following philosopher Jan Patočka (which I will return to below) one can also say that being on the receiving end of the cracking whip of domination creates a sentiment between former adversaries—a *solidarity of the shaken* [46]. Similar to philosopher Rahel Jaeggi, I will call the subjects of this new solidarity *comrades* in order to distinguish it from other forms of solidarity.^4^ In the remainder of this text I will explore what this new kind of solidarity is and how it manifests in a concrete case.

## Solidarity and the Struggle over Welfare Arrangements

Solidarity (in its more conventional form) and the welfare state are tight-knit phenomena. It is therefore reasonable to assume that the dismantling of the welfare arrangements of European states also entails the weakening of solidarity as a principle. Yet, some have identified possible anomalies in this correlation. The Norweigan political scientist Steinar Stjernø, for example, hints at the intimate connection between the contemporary *decomposition* of the welfare state on the one hand, and the formation of solidarity-like bonds between some of its stakeholders that may seep out from the cracks of its neoliberal rearrangement. Firstly, he claims, welfare society has “created” occupational groups that “serve it”,^5^ and consumers that benefit from it, both of which will stand ready to defend the welfare society in times of crisis. Furthermore, alliances between the direct producers and consumers of welfare services may form when these arrangements are contested:

In the struggle over welfare arrangements, strong alliances may develop between occupational groups and professionals serving the welfare state and the consumers of its services and the recipients of its transfers [56]

Nevertheless, Stjernø is quite pessimistic about the potential and stability of these “alliances”. Although similar to solidarity in some sense, alliances fail to qualify as solidarity proper for a number of reasons:

This possible new alliance is more difficult to stabilise and more vulnerable to internal disagreement [...] The professionals who serve the welfare state and its consumers are characterised by a high degree of fragmentation, organisationally and politically. They are not bound together by reciprocal feelings of empathy. They do not share a common vision of society. They do not experience a common adversary that can serve to unite them. As we have seen, these were important characteristics of the ideology of the labour movement in the heyday of working-class solidarity in Scandinavia [56]

There are three important points to be made with respect to Stjernø’s claims. Firstly, Stjernø operates with the assumption that “working-class solidarity” of labor movements is qualitatively different from the “old” solidarity, whose main characteristics have been mentioned above. Secondly, I would like to argue, Stjernø is wrong to claim a priori that the newly formed bonds between (healthcare) professionals and consumers do not express any of the characteristics of what he calls “working-class solidarity”. Thirdly, there is admittedly something special to the “new” solidarity I propose. There is a certain negativity to it, that paradoxically might both qualify and disqualify the phenomenon as solidarity. I will expand on these points.

This is where I turn to the other solidarity, the undercurrent of solidarity tied to emancipatory politics rather than retaining *status quo*. There is indeed a tradition of using the word in a slightly different way, rooted in the rhetoric of the European labor movements, before they followed the Social Democratic turn to a non-conflictual understanding of solidarity in the first half of the twentieth century.^6^ In his monumental tracing of the conceptual history of solidarity in Europe, Stjernø terms this conflict-oriented type “working-class solidarity”, or “Classic Marxist solidarity” [56]. In contrast to the (old) Durkheimian take on the concept of solidarity, whose main objective is social integration, the main objective of marxist solidarity is the realization of group interests. Most importantly, the “common interests” are articulated “against a third party” [56]. Many have noticed the distinctive nature of this “type of solidarity”, and returned to its “permanent characteristic: […] that it involves a commitment *against* an opponent, from whom positive goals must be wrung […] This leads to a *negative* component significantly differentiating this type of solidarity from those mentioned previously” [5]. This type of solidarity has carried additional names (atop of those already mentioned): “oppositional solidarity” [32], “fighting solidarity” [34], “political solidarity” [50], or “solidarity of the shaken” [46]. For many thinkers mentioned above, the main negative component of this other solidarity lies in the way rebellion and conflict against an adversary is the constitutive basis of solidarity. It is through the negativity of rebellion that solidarity in turn is negative. According to Kurt Bayertz, this position has been elegantly summarized by Albert Camus in *The Rebel*: “Man's solidarity is founded upon rebellion, and rebellion, in its turn, can only find its justification in this solidarity”. For Camus, rebellion is for collective and daily life what the cartesian dictum of “cogito” is for solitary being: “I rebel—therefore *we* exist” [10]. On closer inspection, however, the specific concept of solidarity relevant for our purposes consists of not one, but multiple negative components. Beyond the negativity of rebellion, there is the negative character of the common denominator between those who become part of this peculiar (non-)community of solidarity. In the beginning, they share no common vision of society, nor do their “feeling of solidarity” derive from an “identity of class position”, if one is to speak with the words of Friedrich Engels [20]. In order to develop this new concept of solidarity, I turn to a concrete case of what Stjernø would call “struggle over welfare arrangements”: sickness certification behavior of Swedish doctors in austere times.

## Case Study: New Solidarity Between Disempowered Swedish Physicians and Patients in the Sickness Certification Process

The Swedish social security system provides universal income protection in case of inability to work due to sickness. After one week of sick leave, patients are required to provide the Swedish Social Insurance Agency (SSIA) with a sickness certificate, which is compiled by their treating physician, often a general practitioner (GP) at a primary health center. In the last instance it is the SSIA that decides eligibility for sickness benefits. The SSIA can accept the certificate as is, ask for further specification or reject it outright [55]. In recent years, the SSIA has become more rigorous in assessing eligibility of patients, thereby limiting the clinical discretion of physicians in this regard [16, 22, 52]. For example, between 2014 and 2016 the proportion of rejected sickness certificates increased threefold [23]. The Swedish state is certainly not unique in narrowing the scope of social insurance in general, and sickness benefit systems in particular, in this way [25]. This restructuring of social insurance systems, often labelled “neoliberal”, is a subset of the rearrangement of the welfare state as a whole (discussed above).

This cluster of reforms has had far reaching consequences for both patients and doctors. For the patients, rejected sickness benefit claims can have serious economic consequences, not to mention the negative health related consequences of the two undesired outcomes of being forced to work despite debilitating sickness, or having no income because of a debilitating sickness. Doctors have reported numerous problems with this rearranged determination of eligibility for sickness benefits. GPs belong to one of the medical specialties that deal with sickness certificates on an almost daily basis, and have been particularly vulnerable to the recent changes: between 2004 and 2017, the proportion of GPs who reported that their medical judgments were questioned by the SSIA rose from 10 to 57%. The number of GPs who experienced that the SSIA requested unnecessary corrections to the sickness certificates increased from 48 to 72% between 2012 and 2017. In 2017, 72% of all surveyed GPs felt that the SSIA requested “objective findings” in cases where objective signs are notoriously difficult to identify in the clinical setting (e.g. psychiatric disability, chronic pain, etc.) [3]. Hence, for doctors, the changes have resulted in two types of problems: their medical professional autonomy and discretion is questioned and curtailed, and their working conditions have deteriorated by the increased paperwork required by the SSIA.

The ideological and paradigmatic underpinning of these reforms has been visible both in the public debate as well as the research on sickness certification praxis. The Swedish public discourse on sickness shifted during the 1990s, and the problem of sickness absence was reframed from being mainly a public health concern, to a problem of malingering patients and illegitimate overuse of a so-called “too generous” sickness benefit system [31]. Sick patients were hence implicated as malingerers taking advantage of their doctors, the healthcare system and the sickness benefit system. Similarly, the thematics emerged in European research on doctor–patient dynamics regarding sickness certification. People were forcing their will to receive sickness benefits on doctors, and the doctors were passively yielding to their demands [28]. In fact, the majority of studies conducted on sickness certification praxis focused on the doctor–patient relationship as *primarily conflictual* as well as *the primary conflict* in sickness certification [43, 44, 65].

### Everyday Resistance Against the Sickness Benefit System

The processes delineated above could certainly be interpreted as expressions of a general loss of trust and solidarity, which have been superseded by a universal logic of suspicion [40, 61]. It could easily be assumed that doctors follow suit and act equally suspicious towards their patients and their sickness benefit claims. However, that does not seem to be the case. Physicians are not just standing idly by. Some of them mount elaborate forms of low-key everyday resistance against the attempted power grab of the SSIA, its stricter standards of sickness benefit eligibility, its logic of austerity and suspicion. One such way of resistance has been documented through interview studies with Swedish GPs. In writing the sickness certificate, they employ “techniques” aimed at making the certificate appear as if it adheres to the stricter standards. For example, as the SSIA demands documentation of “objective signs” for cases where such signs are hard to come by (such as psychiatric disease, some forms of chronic pain, etc.), the appearance of such data is fabricated on the fly. Hence, parts of the anamnesis (the patient’s own account) might be presented *as if* it was an objective finding: an account of a depressed mood is “translated” to a clinical sign (“patient has a lowered mood” or “psychomotor retardation”). In the interview study, the motives declared by physicians for doing so were multiple: writing sickness certificates in an evasive fashion was not motivated solely by self-interest, nor by pure altruism. The doctors saved time, so they could do more clinical work instead of paperwork, and defended their medical autonomy and discretion. The patient could recuperate and enjoy income protection [52, 53].

In both the experience of and resistance against the bureaucratic disregard for professional judgment, the *absurdity*—that is, the contradictions and senselessness—of bureaucracy appeared in between the lines of the interview study of Swedish GPs. For example, one doctor lamented that the SSIA forced her to use buzzwords which paradoxically lowered the medical precision of their certificates:

I write ‘cognitively impaired’, because I’ve learnt that they [the SSIA] want to hear that particular phrase. It’s not enough to write memory and concentration loss; for some reason the word ‘cognitive’ must be used [52]

Furthermore, this perceived absurdity was commonly communicated between the doctor and patient. The doctor could be frank with the patient about the weirdly Kafkaesque and common predicament of theirs, exemplified here:

I’ve even told the patient: “Maybe this wasn’t exactly what you told me, but to be certain that you’ll get your sick pay, I will have to write it like this.” [52]

### The “New” Solidarity of Disempowered Comrades

The dynamics between doctors and patients in the sickness certification process, the transition from adversaries to comrades in and through their common powerlessness, can be understood in terms of philosopher Jan Patočka’s idea of a “solidarity of the shaken”.^7^ In an essay in his *Heretical Writings* (“Wars of the Twentieth Century and the Twentieth Century as War”) Patočka develops the idea that the horrors in the trenches of the First World War revealed something very important regarding human coexistence. Through depictions of the front-line experiences, he highlights a special type of coexistence, enabled by the limit-experience of war, of death, and violent conflict. The experience was utterly destabilizing and frightening: “The first phase, which few can transcend, is the experience of meaninglessness and unbearable horror. The front line is absurdity *par excellence*”. One of the main consequences of this fundamental shaking is that the “adversary”—the one with whom we have nothing in common but conflict—becomes a “fellow participant in the same situation”, through the absurdity of war, and through the brush with death. This is what Patočka calls “the solidarity of the shaken for all their contradiction and conflict”. Whichever trench we happen to crouch down in, German or British, in the role of doctor or patient, we come to the insight that *“*ultimately, all are subject to the crack of the whip*”* [46].

Likening mundane clinical encounters to life in the trenches of war may seem implausible, even tasteless. However, metaphors of war are commonplace in portrayals of healthcare, even though their use can be problematic. It has been claimed that they stigmatize the ill and shift focus “from fighting the disease to fighting the patient” [42]. Yet, there are some fundamental similarities between war and the clinical encounter that warrant a comparison aided by Patočka’s *solidarity of the shaken*. The concept constitutes a *critique* of the senseless killing of war and is therefore less prone to reproduce the negative impact of warfare tropes; it might even counter such effects. The three fundamental similarities are the following: (1) Both war and the clinical encounter can involve severe suffering and death. Through illness as the reminder of the finitude of life, death figures at least to some extent in the background of many clinical encounters. Havi Carel, an influential thinker in the field of phenomenology of illness, goes further and establishes death as a transcendental-existential condition of illness as such: “Death is positioned on the existential horizon of illness, and therefore makes up part of the illness experience” [12].^8^ Furthermore, for the doctor, there is the limit-experience of being overworked to the brink of fatigue. It is a form of “self-sacrifice” and an “*endurance* in the face of death”, not exclusively in relation to the patient, but also through an ever accelerating production process [46]; (2) Both war and clinical encounters in the era of bureaucracy are in some respects *absurd*: In war, the horror of death is its necessary condition. In healthcare, this may occasionally be the case. There is, however, another dimension of existential loss of meaning or ground that springs forth not from facing death in itself, but from confronting nightmarish bureaucratic inflexibility. This holds true for war as well: while the horror of death is necessary, it is not a *sufficient* cause of that specific type of absurdity. Rather, it is the horror of death *in conjunction with* the destruction of human bodies on an unprecedented industrial scale, initiated by impersonal state structures and carried out by opaque chains of command. Hence, the absurdity in question is not only of the transcendental Sisyphean variant, but simultaneously a worldly and historically specific subspecies often emphasized in the works of Franz Kafka.^9^ When internal ends of healthcare are increasingly colonized and replaced by external goals of efficiency and austerity, a similar kind of absurdity emerges, that may lead to losing and being forced to find new ground. In the case of the absurdity of the sickness certification process (mentioned above), that new ground is the solidarity between physicians and patients; 3. There is a procedural similarity between the solidarity of soldiers in war and the encounters between doctors and patients in late capitalism. In contrast to the solidarity of, say, factory workers who start out from a relation of mutual *indifference* to showing solidarity with each other, the starting point of this other form of solidarity is not indifference, but *antagonism*, from which they transition over to solidarity.^10^ In a way, the foundations of this new kind of solidarity were built by the metaphors and models of partnership and consumerism. The abandonment of the paternalist paradigm also meant that the interests of physicians and patient could not be assumed to automatically overlap. In the superseding framework of partnership their interests often appear as subject to negotiation, and in the framework of consumerism the participants even appear as mutually suspicious adversaries, whose interests must not only be negotiated, but sometimes even fought for [24]. Antagonism is essential to market mediated relations, even if they do not always appear as such. Irrespective of the use-value of whatever is changing hands, the goal is nevertheless for one to buy cheap and for another to sell high. However, the argument of a foundational antagonism between doctor and patient is independent of the conceptual content of the partnership and consumer models. For example, the relationship between doctors and patients in the sickness certification process does not embody any specific doctor–patient relationship model. Even if the logic of mistrust pervades the entire field, in Sweden it is not directly a commodity relation because the *Social Insurance Agency* is state-run and can only indirectly be understood in terms of the consumer model. Due to the institutional framework, the doctor and the patient have still been cast into the pit of sickness certification as adversaries, as “gatekeeping” the resources of the social insurance system is one of the main roles that the Swedish doctor is expected to assume [60]. Given current circumstances, that role unfortunately amounts to being the guardian of austerity [4].

In addition to these points, which make Patočka’s (ontologically) negative take on solidarity relevant for our purposes, the concept is also distinctly negative in one further sense. Whereas Steinar Stjernø believes that the lack of “a common vision of society” between physicians and patients automatically disqualifies their relation as being solidaristic, this apparent lack is an essential feature of Patočka’s solidarity: “The solidarity of the shaken can say ‘no’ to the measures of mobilization which make the state of war permanent. It will not offer positive programs but will speak, like Socrates' *daimonion*, in warnings and prohibitions” [46].

Hence, the Patočkean take on solidarity, a subspecies of non-Durkheimian oppositional solidarity, extends the negative character of the phenomenon. In *addition* to being negative by being grounded in rebellion and opposition to an adversary, it is also negative insofar as it unites through the negative experience of death and suffering; that the point of departure for those who solidarize with each other is discord; and finally that it does not offer a “positive program” or have a “vision of society” common to those who are shaken.

### Solidarity of the Shaken Physician and Patient in the Sickness Benefit System

It is here time to critically revisit Steinar Stjernø’s claim that the alliance between professionals serving the welfare state and their clients (in our case doctors and patients) fails to qualify as “solidarity” proper. It might be true that the phenomenon does not qualify specifically as “working-class solidarity”, as physicians and patients do not necessarily “share a common vision of society”. However, as seen above this should not automatically disqualify the phenomenon as solidarity. For Jan Patočka, this positive program is not required for a “solidarity of the shaken”. Hence, the concept of solidarity might be salvaged if one reconfigures its meaning. It is also noteworthy that Stjernø’s postulated lack of “a common adversary that can serve to unite them” is simply not true in the case study. The common adversary, holding the whip of austerity, is the SSIA. Furthermore, it is the very same whip that redraws the lines of conflict. Doctors and patients start off as adversaries in the sickness certification process, their interests are pitted against each other. Nevertheless, being shaken, simultaneously by the whip of austerity and the encounter with the *absurdity* of bureaucracy, aligns them. The solidarity is evident in the forms and motives of covert resistance practices employed by the physicians. This latter form of negativity (of resistance) is not unique to Patočka’s concept of solidarity but is common to the family of “solidarities” to which it belongs.

## Conclusion and the Question of Generalizability

In terms of power, the many metaphors of the doctor–patient relationship can be clustered into three distinct groups: paternalism (in which the doctor wields power over the patient); partnership (in which doctor and patient shares the power); consumerism (in which the patient wields power over the doctor). By ordering the metaphors in terms of power and temporal ordinality, a *fourth* possibility arises: a situation in which *neither* doctor *nor* patient have any significant power.

This mutual powerlessness creates a special type of bond between doctors and patients. I have argued that they relate to each other as *comrades*, and that an adequate descriptor of the properties of this relationship is *solidarity* (of the shaken). Rather than social integration, solidarity signifies the unity of those who “are subject to the [same] crack of the whip” [46].

The case study demonstrates the dual effects of austerity: It alienates both doctor and patient, rendering them both powerless; at the same time, through the very same process, it creates the very conditions of its own potential undoing. In the Swedish case, the narrowed eligibility criteria for sickness benefits (a consequence of austerity logics) forces doctors to abandon the impossible task of reconciling their “dual roles” as patient advocates and representatives of the authorities, and side with their patients. The “horror” of being pitted against their patients turns them into allies, and even establishes a form of negative solidarity through this common experience of horror and stress brought about by austerity, bureaucracy and institutional powerlessness. This has warranted the introduction of the concept of “the solidarity of the shaken”, devised by Jan Patočka.

An important question is if this “solidarity of the shaken” is generalizable. Can it really be considered somewhat paradigmatic? The idea and phenomenon of (infra-)political *solidarity* between doctors and patients that transcends the traditional therapeutic alliance between them has actually gained some traction in recent years. However, this kind of solidarity has almost exclusively been observed in times of intensified political conflict: On *Tahrir Square* in Cairo during the Arab Spring in 2011, doctors in makeshift tent facilities and protestors forged a deep form of alliance in and through the common adversary, repression and horror they faced [26]; In 2013, the infirmaries in *Gezi Park* in Istanbul “acted as new networks of solidarity and comradeship among doctors and protestors” [11]; Another recent but less militant example of the same phenomenon was observed in the battle against privatization and market-based administrative ideologies of the British NHS, and the political attacks on the working conditions of junior doctors which culminated in a series of labor strikes in 2016 in the UK. Ranajit Pushkar observed that the “moral and practical logic” of NHS activists and doctors aligned the duties and interests of health care workers and health care users, effectively making possible the formation of a “new” type of “solidarity” between them, based on the recognition of their “mutuality of interests” [47].

What is really at stake in the choice of seemingly descriptive terms? Does it matter if the phenomena above are called “alliance” or “solidarity”? There is of course a normative aspect at play here. Alliances are fleeting and temporary. By arguing for solidarity, the depth and significance of the mutual interests and sufferings of doctors and patients are brought to the fore.

## Footnotes

1 Other models suggested are quite similar, if not identical, to this so-called consumer model. For example, what has been called the *informed model* [14] shares many of the attributes of what I will discuss below.

2 The idea that consumerism empowers consumers (patients) requires one important qualification. Whether or not it really empowers the consumer depends on which “dimension” of power one is referring to. If power is to be understood as “decision-making” (which I do throughout the article), then it is indeed possible to say that consumers wield the formal power and freedom to choose this or that commodity as they see fit (given that the market functions in the way it is supposed to by providing the consumer several alternatives to choose between). However, power can also be understood as a multi-dimensional phenomenon (as suggested by for example Steven Lukes), constituted not only by “decision-making”, but also by power as “control over the agenda”, as well as control over the “perceptions, cognitions and preferences” of others [37]. The agenda of (or the scope of options on) the market is less directly controlled by the consumer. That power in consumer society resides elsewhere than it may appear at first sight, in the sense that it might entail power in the first dimension (freedom of choice), but disempowerment in the second and third, has been thematized by for example scholars in the tradition of critical theory during the twentieth century, such as Herbert Marcuse and Erich Fromm [33]. There are also studies of the aforementioned ambivalence of consumerism in healthcare, and the exercise of the third kind of power that shapes the very desires and preferences of patient-consumers [29, 51].

3 Goodyear-Smith and Buetow hint at a particular experience springing forth from a “shared weakness” in the face of the clinical inability to cure a disease. This powerlessness is directed toward natural, rather than social, fact [24]. This article deals with the latter.

4 “We can see the significance of the distinction between solidarity and compassion when we look at the long history of oppressed groups calling for (and building on) solidarity. Obviously, being treated as a 'comrade' as opposed to being treated as a helpless victim affects the self-esteem of those concerned” [30]. In Sweden in particular, and in Europe in general, one colloquial use of the word “solidarity” tends to dominate. It denotes a welfare arrangement in which “everyone is assumed to make a fair financial contribution to a collectively organized insurance system that guarantees equal access to health and social care for all members of society” [61]; also see [38, 41]. When talking about solidarity in this way, the community in question is not just any community, but society as a whole. There are of course many different takes on the characteristics of this solidarity-cum-social cohesion. Despite a considerable heterogeneity, there is something that remains invariable throughout its use in classical sociology and the political discourse of both Social Democracy and Christian Democracy: solidarity is the opposite of *conflict*, which in turn is banished from playing any part in the concept [56]. Jacques Donzelot has written a short history of the co-optation of the concept of solidarity during the inception of the Third Republic in France that robbed it of its conflictuality [17]. David Lockwood has argued for abandoning the concept of solidarity, as it is hopelessly tied to sociological functionalism of Émile Durkheim [36].

5 Among these “large number of occupational groups” that have been “created” by the welfare society are, according to Stjernø, physicians. Although the claim that the profession of medicine was outright *created* by the welfare state might be contested (it was for a long time independent from it), its social position was certainly regulated, modulated and consolidated in different ways by the welfare state. For a Swedish example of these processes, see [13, 21].

6 Hence, it should be admitted that the term “new” might be misleading, as there are roots that go further back.

7 This “solidarity of the shaken” is part of a greater, yet non-systematic, philosophical exploration the "three fundamental movements of human life" [46], which bear similarities both with Heidegger’s existential analysis of time and Hannah Arendt’s partition of the spheres of human life into labor, work and action [49, 59]. It should be admitted that I do not do justice to the totality of Patočka’s thought. My aim is here not to expand on Patočka’s thought in general, but to operationalize his interesting take on the idea of solidarity.

8 Havi Carel’s claim is certainly controversial. It can be argued that there are healthcare encounters where suffering from illness, let alone death, is no more than a hardly distinguishable point in the distance. Medical encounters devoid of any suffering are difficult to imagine (perhaps routine check-ups, that nevertheless involve previous or potential future suffering). There are indeed cases where *serious* harm or death are not prominent at all: cosmetic interventions, routine check-ups, and an infinite number of benign health concerns. On the other hand, there are exceptions to the association between death and war as well (not everyone fought in the frontline). Nevertheless, I believe the *general* claim that war and healthcare are associated with death (specifically with the production and prevention of death, respectively) is valid.

9 For Albert Camus, one of Kafka’s great contributions consisted of identifying and presenting the absurd as encompassing *both* dimensions (the “metaphysical” or “supernatural” versus the secular, historical, worldly or “everyday” dimension) at the same time [9].

10 Indifference and antagonism as points of departure for different kinds of solidarity should be understood as ideal types. Empirically, there is considerable overlap. Even in the case of factory workers, antagonism may be present from the very beginning: the race to the bottom of unorganized labor where each worker undercuts the other with lower wages, scabs replacing workers on strike, as well as the stratification of workers in terms of other axes of domination (gender, race, etc.). However, antagonism is not a necessary part of the type of solidarity of factory workers. They solidarize despite, not because, of the antagonism.
